# Differential Patterns of Planning Impairments in Parkinson's Disease and Sub-Clinical Signs of Dementia? A Latent-Class Model-Based Approach

**DOI:** 10.1371/journal.pone.0038855

**Published:** 2012-06-08

**Authors:** Lena Köstering, Audrey McKinlay, Christoph Stahl, Christoph P. Kaller

**Affiliations:** 1 Department of Neurology, University Medical Center, University of Freiburg, Freiburg, Germany; 2 Freiburg Brain Imaging Center, University of Freiburg, Freiburg, Germany; 3 Department of Psychology, University of Canterbury, Christchurch, New Zealand; 4 School of Psychology and Psychiatry, Monash University, Melbourne, Australia; 5 Department of Psychology, University of Cologne, Cologne, Germany; Technical University of Dresden Medical School, Germany

## Abstract

Planning impairments mark a well-documented consequence of neurodegenerative diseases such as Parkinson's disease (PD). Recently, using the Tower of London task we demonstrated that, rather than being generally impaired, PD patients selectively fail when planning requires flexible in-breadth search strategies. For a better understanding of the interindividual patterns underlying specific planning impairments, here we performed an explorative re-analysis of the original data using a latent-class model-based approach. Data-driven classification according to subjects' performance was based on a multinomial processing tree (MPT) model accommodating the impact of increased breadth versus depth of looking ahead during planning. In order to assess interindividual variability in coping with these different task demands, an extension of MPT models was used in which sample-immanent heterogeneity is accounted for by identifying different latent classes of individuals. Two latent classes were identified that differed considerably in performance for problems placing high demands on the depth of anticipatory search processes. In addition, these impairments were independent of PD diagnosis. However, latent-class mediated search depth-related deficits in planning performance were associated with poorer outcomes in dementia screenings, albeit sub-clinical. PD patients exhibited additional deficits related to the breadth of searching ahead. Taken together, results revealed dissociable impairments in specific planning processes within a single task of visuospatial problem solving. Present analyses put forward the hypothesis that cognitive sequelae of PD and sub-clinical signs of dementia may be related to differential patterns of planning impairments.

## Introduction

Parkinson's disease (PD) is a neurodegenerative disorder typically characterized by motor symptoms such as bradykinesia, rigidity, and resting tremor. In addition, cognitive impairments are present, even in early disease stages, and predominantly affect executive functions such as planning abilities [1], [2]. As a case in point, several studies reported impairments of PD patients in visuospatial planning on the Tower of London (ToL) task (e.g., [3], [4]). However, the exact nature of planning impairments on the ToL remained unclear. Previous studies suggest that PD pathology might only affect planning latencies but not accuracy [5], [6], and it might not significantly affect patients' performance until progression to severe PD [6]–[8].

Recently, it has been proposed that impairments of PD patients in various cognitive tasks can be explained by a common deficit in cognitive flexibility [1]. According to this model [1], [9], the stability and flexibility of cognitive representations is related to the transmission of prefrontal and striatal dopamine (DA), respectively. The flexible adaptation of mental representations to environmental or task demands relies on phasic activity of DA in the striatum [10], [11]. In contrast, the stability of representations, that is, their maintenance over a period of time in the presence of distracting or irrelevant stimuli, is associated with tonic DA levels regulated by prefrontal dopaminergic activity [10], [11]. As PD pathology primarily leads to a degeneration of dopaminergic nigrostriatal projections [12], [13], it is assumed to impede phasic DA activity in the striatum, thus provoking deficits in cognitive flexibility [1]. In accordance with this, PD patients have been shown to exhibit impaired performance in paradigms taxing cognitive flexibility such as attentional set shifting and task-switching (e.g., [6], [14]; for a review, see [1]).

**Figure 1:**
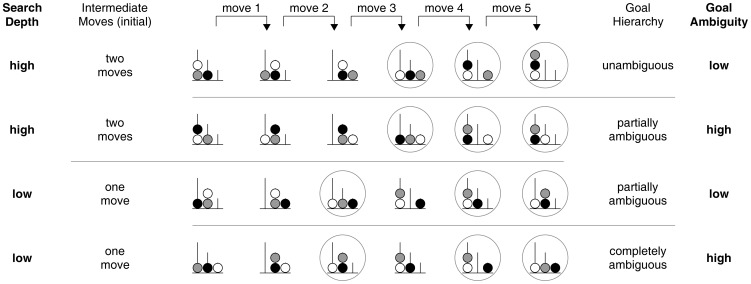
Experimental design by McKinlay et al. [15]. In five-move Tower of London problems, two predominant structural patterns are evident leading to either low or high demands on *Search Depth* with either one or two initial intermediate moves, respectively (cf. [45]). Despite three different levels of goal hierarchy (problems with tower, partial tower, or flat configuration leading to unambiguous, partially ambiguous, or completely ambiguous goal hierarchies; cf. [45]), not all possible combinations with *Search Depth* are existent in the Tower of London problem space. The different levels of goal hierarchy were therefore hierarchically nested under the levels of *Search Depth*, resulting in the factor *Goal Ambiguity* featuring two levels with high and low demands (see also [15]). Consequently, the experimental design comprised a 2×2 factorial manipulation of *Search Depth* and *Goal Ambiguity*. Circles around states denote goal moves.

Given the strong empirical support for deteriorated flexibility of cognitive representations in PD, we recently investigated whether planning performance of PD patients is sensitive to differential requirements for flexible search strategies during planning [15]. The demand on flexibility and stability of search processes was manipulated through systematic variations in goal hierarchy and search depth of ToL problems, respectively (Fig. 1). Although there might be also some overlap between the cognitive demands imposed by these two structural problem parameters (cf. [15]), goal hierarchy and search depth can be seen as placing higher requirements on the breadth versus the depth of look-ahead search processes, respectively. In detail, ambiguous goal hierarchies do not provide a clear action sequence [16]; instead, they require a broad search amongst several move alternatives so as to establish the optimal sequence of final moves, thereby putatively taxing processes of cognitive flexibility. For instance, if all balls of the goal state are stacked on a single rod, the ball at the bottom definitely has to be in its goal position before the ball that is second from the bottom and so on. In contrast, if the three balls are distributed across the three rods of the goal state, no information about the sequence of the final moves is provided and it has be to identified by look-ahead search with emphasis on in-breadth search processes. In contrast, the search depth of a problem determines the number of intermediate moves that have to be considered before the first goal move [16]. This entails generating a succession of intermediate moves while taking into account their interdependencies. That is, the higher the search depth of a problem, the more successive intermediate moves and resultant interdependencies have to be anticipated. Therefore, higher search depths place an increased load on the depth of look-ahead search processes and thereby possibly on cognitive stability. In the study of McKinlay et al. [15], it was found that PD patients did not differ from age-matched healthy controls in overall accuracy levels, but that they solved significantly fewer problems with high goal ambiguity. As no interaction of PD diagnosis was found with search depth, these results argue for a selective impairment of PD patients in dealing with ambiguous goal hierarchies that place increased demands on the breadth of searching ahead [15].

These findings were based on classifying participants according to membership in a manifest group (i.e., PD diagnosis versus healthy controls) and testing for between-group differences in the ability to deal with the varying demands on the flexibility and stability of looking ahead [15]. By extension, such an approach assumes homogeneity of the underlying cognitive processes related to the breadth versus depth of planning across all participants of a manifest group [17]. However, flexibility and stability of cognitive representations are also subject to interindividual variability in phasic and tonic dopaminergic activity independent of pathological processes [9], [10]. Therefore, within-group homogeneity of the cognitive look-ahead search processes associated with increased demands on flexibility and stability cannot be assumed. On the contrary, it is highly likely that even in a sample of PD patients other factors than PD pathology influence planning abilities and further contribute to systematic variation in performance of search processes during problem solving (cf. [3]).

Recent methodological advances enable researchers to investigate this heterogeneity. Here, we present an explorative re-analysis of the data of McKinlay et al. [15] using a data-driven approach that examines the presumed sample heterogeneity in cognitive look-ahead processes based on participants' individual planning performance. That is, instead of classifying participants according to clinically or theoretically derived manifest characteristics and then testing for differences in performance, the latent-class model-based approach adopted here follows an opposite logic: First, participants' planning performance in responding to varying demands on the depth and breadth of planning processes is modeled using a multinomial processing tree (MPT) model [18]. In a second step, sample heterogeneity is assessed by directly testing whether interindividual differences in the modeled cognitive processes exist, and if so, whether they can be accommodated by subdividing the overall sample into sets of latent classes [19], [20]. Based on this data-driven classification, it is then possible to test if membership in different latent classes – and, thereby, differences in cognitive processes underlying planning performance – is associated with differences in manifest characteristics such as PD diagnosis.

Following this explorative approach, present re-analyses revealed an unexpected classification of participants along dimensions of varying depth of look-ahead processes that (i) appeared to be orthogonal to PD diagnosis, and, most intriguingly, that (ii) was associated with poorer outcomes on diagnosis and screening instruments for dementia. Often reported planning disturbances in PD and dementia may hence have different cognitive origins that are dissociable within a single task.

## Materials and Methods

As this study constitutes a re-analysis of data previously reported by McKinlay et al. [15], only information necessary for understanding the present results will be briefly summarized here. For a more detailed overview on the applied materials and methods of the original study, please refer to the comprehensive descriptions provided in McKinlay et al. [15]. Assessments were carried out at the University of Canterbury (NZ) and the study protocol was approved by the Canterbury Ethics Committee.

### Subjects

Thirty non-demented and non-depressed patients with idiopathic Parkinson's disease (PD) were assessed in the study of McKinlay et al. [15]. PD was diagnosed by a neurologist who specialized in movement disorders. Mean age at onset of PD was 57.17 years (SD = 8.75), mean disease duration was 7.33 years (SD = 4.57). All patients were on anti-Parkinsonian medication and were tested while on optimal levels (11/30 dopamine agonists; 1/30 selective MAO-B inhibitors; 8/30 dopamine agonists and anticholinergic agents; 4/30 dopamine agonists and MAO-B or COMT inhibitors; 3/30 anticholinergic agents and MAO-B or COMT inhibitors; 3/30 dopamine agonists, anticholinergic agents, and MAO-B or COMT inhibitors). Thirty healthy controls were individually matched in terms of age and pre-morbid intelligence.

Inclusion criteria concerned age (between 50 and 80 years), English as primary spoken language, adequate or corrected hearing and vision and, for PD patients, a Hoehn and Yahr [21] rating of stage I-III. Exclusion criteria concerned any history of moderate or severe head injury, stroke or other neurological impairment, major medical or psychiatric illness, current involvement in therapeutic trials, suspicion of dementia (Mini Mental Status Exam [22], MMSE<25), pre-morbid IQ<85 (as assessed with the National Adult Reading Test, NART [23]), acute depression (Beck Depression Inventory-II [24], BDI-II>17) or major depressive episode in the previous six months, and taking in any other than anti-Parkinsonian medication known to have significant effects on the central nervous system.

### Experimental task and paradigm

Planning ability was assessed using a computerized version of the Tower of London (ToL) task that was originally developed to measure planning impairments in frontal lobe patients [25]. In the ToL, planning is required for an efficient transformation of a given start state into a desired goal state, that is, for an optimal solution within the minimum number of moves. The task's general scenario is knowledge-lean and well-defined with explicit specification of the start and goal state, the transformation operators, and their restrictions [26]. The classic version of the ToL consists of three differently colored balls placed on three vertical rods of different heights that may hold at maximum one, two, or three balls, respectively.

Start and goal states were presented in the lower and upper half of the screen, respectively. Subjects were instructed to transform the start state into the goal state while following three rules: (1) only one ball may be moved at a time; (2) a ball cannot be moved while another is lying on top of it; and (3) three balls may be placed on the tallest rod, two balls on the middle rod, and one ball on the shortest rod. Subjects were instructed to solve each problem in the minimum number of moves (indicated on the screen). To match the goal state, subjects had to operate on the start state. Movements were executed on an ELO 17″ touch sensitive screen. Individual trials were initiated by the experimenter. Before displaying the next problem, subjects were prompted by the program to plan ahead first.

The assessment of planning ability occurred in two parts. Present re-analyses, however, concern only the second part that addressed more complex planning demands in a set of five-move problems. The specific aim was to disentangle the contributions of two distinct aspects of ToL problem structure, that is, search depth and goal hierarchy, to planning impairments, while the minimum number of moves was kept constant (Fig. 1). Due to general features of the ToL problem space, the combination of both search depth and goal hierarchy inevitably results in an imbalanced design since certain problem configurations do simply not exist. Testing for possible interactions between goal hierarchy and search depth would therefore be unfeasible [17]. However, to allow for a factorial analysis of the interesting main effects and interactions with group, McKinlay et al. [15] transformed the composition of the two structural problem parameters into a hierarchical design by nesting the relative ambiguity of goal hierarchy, i.e., *Goal Ambiguity*, under the levels of *Search Depth* (Fig. 1).

The resulting 2×2 design included hence a factorial manipulation of *Goal Ambiguity* (high vs. low) and *Search Depth* (high vs. low). Two problems per cell were presented. The number of problems correctly solved in the minimum number of moves served as dependent variable.

### Classification using a cognitive model for separating different planning demands

Data-driven classification according to subjects' performance was based on a multinomial processing tree model with latent classes [19], [20]. Multinomial processing tree (MPT) models are a specific family of models in the general class of parameterized multinomial models [18]. MPT models are tailored to specific experimental paradigms; their parameters represent probabilities of latent cognitive processes. They are widely used as measurement models in cognitive psychology (for a review, see [27]).

**Figure 2:**
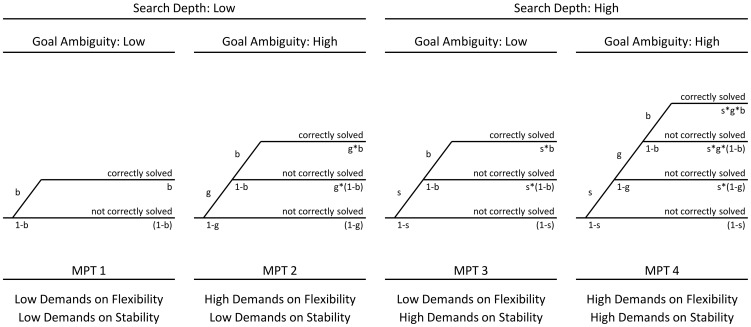
Multinomial processing trees (MPT) of the current model of planning performance. Based on the four resulting cells of the hierarchical 2×2 design applied by McKinlay et al. [15], the present model comprised four independent multinomial processing trees (MPT) in total. The four trees spanned the baseline parameter *b* as well as task-demand parameters *f* and *s*. More specifically, paramaters *f* and *s* reflected additional cognitive demands on the breadth and depth of planning processes, respectively, and were imposed by higher levels of *Goal Ambiguity* (*g*) and *Search Depth* (*s*).

Here, an MPT model based on the hierarchical design applied in the study of McKinlay et al. [15] was used to disentangle different demands on the breadth versus depth of planning processes as imposed by the experimental manipulations of *Goal Ambiguity* and *Search Depth*, respectively (Fig. 2). Performance in problems with low demands on both parameters served as baseline that is represented by the parameter *b*. The level of additional cognitive demands evoked by a higher *Goal Ambiguity* was measured by the parameter *g*, which is expected to be 1 if an increase in *Goal Ambiguity* does not affect performance at all. Otherwise, it is expected to be less than 1 depending on the actual degree by which higher levels of *Goal Ambiguity* exert detrimental effects on planning performance. Likewise, the level of additional cognitive demands evoked by a higher *Search Depth* was implemented by the parameter *s* (i.e., it is expected to be 1 if an increase in the level of search depth does not affect performance, and is expected to be less than 1 depending on the degree to which higher search depths exert detrimental effects on planning accuracy). Taken together, the model has three parameters: baseline performance was reflected by parameter *b*, whereas parameters *g* and *s* assessed subjects' abilities to cope with higher demands on the breadth versus depth of searching ahead, that is, higher levels of *Goal Ambiguity* and *Search Depth*, respectively. Based on the two levels (high vs. low) for each of the two manipulated factors, the model consequently comprised four multinomial processing trees (MPT) that reflected the different combinations of planning demands (see Fig. 2). The basic idea of the modeling approach is to test whether subjects' performance can be accommodated satisfactorily by the model parameters. Given the four independent cell frequencies (percent correct for each cell of the 2×2 factorial design, see above), one degree-of-freedom was available for testing the model's goodness-of-fit.

To further account for variability across individuals, a latent-class extension of MPT models was used in which parameter variability is accommodated by identifying different latent classes of individuals [19]. That is, it was explicitly tested whether presumable sample-immanent heterogeneity was better accounted for by a solution with one versus two or more latent classes. Using the HMMTree software [20], maximum-likelihood estimates and indices of model fit were computed for models with different numbers of latent classes. To enable additional analyses (see below), groups were created on the basis of the latent-class parameter estimates. Specifically, posterior probabilities of latent-class membership were used to classify individuals as belonging to one of the latent classes (i.e., individuals were assigned to the latent class for which they had the highest posterior probability).

### Additional analyses

Further exploration of between-group differences for the resultant latent classes was based on tests related to the aforementioned inclusion/exclusion criteria and additionally available information from clinical assessments. Taken together, latent classes were compared concerning subjects' age, years of education, crystallized intelligence (as assessed with the NART [23]), depression scores (BDI-II [24]), the Mini Mental Status Exam as a screening test for dementia (MMSE [22]), age-corrected scores (AMSS) of the more comprehensive Dementia Rating Scale (DRS-2 [28]) and scores on the CLOX Executive Clock-Drawing Test [29] as a measure of executive impairment (the CLOX1 measures executive deficits by demanding unprompted drawing of a clock, whereas the CLOX2 copying task assesses visuospatial abilities). Group assignments among PD patients was also explored with respect to differences in the severity of PD symptoms as assessed by the Hoehn and Yahr [21] rating and the motor scale of the Unified Parkinson's Disease Rating Scale (UPDRS [30]) as well as to differences in age at disease onset, disease duration, and pharmacotherapy.

## Results

### Latent-class modeling and resultant classification

Patients and healthy controls were classified according to their planning performance using a latent-class approach for multinomial processing tree (MPT) models [19]. Based on an MPT model accommodating the impact of *Goal Ambiguity* and *Search Depth* on planning (Fig. 2), maximum-likelihood estimates were computed for one and two latent classes using the HMMTree software [20].

First, general model fit was evaluated for the one-class model, using the goodness-of-fit statistics *M_3_* and *S_1_* suggested by Klauer [19] that are both asymptotically distributed as *χ^2^*. *M_3_* assesses the deviation of the observed cell counts from those predicted by the model, whereas *S_1_* is based on the direct comparison between the observed and the predicted variance-covariance matrices [19]. The tests were capable of detecting small-to-medium deviations by Cohen's [31] convention. Power analyses yielded the following critical *χ^2^* values for *α* = *β* = .01: for df = 1, *χ^2^_crit_* = 6.63; for df = 3, *χ^2^_crit_* = 11.34; for df = 7, *χ^2^_crit_* = 18.48.

Although the one-class model satisfactorily captured the pattern of cell frequencies aggregated across participants (*M_3_*_(*df* = 1)_ = .195<*χ^2^_crit_*, *n.s.*), the respective variances and covariances were not adequately accounted for (*S_1_*_(*df* = 7)_ = 26.04>*χ^2^_crit_*, *p*<.001). A substantial improvement could, however, be gained for the solution with two latent classes that indicated an acceptable replication of the observed data by the model (*S_1_*_(*df* = 3)_ = 9.36<*χ^2^_crit_*, *n.s.*). In other words, sample-immanent heterogeneity could be much better accommodated by a model assuming two latent classes. The assumption that the same set of parameters described the data of each subject equally well was hence unwarranted for the present sample.

Further analysis of the two-class model revealed that this variability was due to interindividual differences in coping with higher demands on the depth of anticipatory search processes: The data were found to consist of a larger class (SD+; comprising 73 percent of the sample) that showed only a moderate loss of planning accuracy in problems with a high *Search Depth* (i.e., the parameter estimate of *s_SD+_* = .78 indicates that performance in problems with higher search depth reached approximately 80% of baseline performance), whereas the smaller class (SD−; 27 percent) indicated with *s_SD−_* = 0 a dramatic drop in performance (i.e., the parameter estimate indicates that performance in problems with higher search depth was at floor levels). An equality restriction for parameter *s* across latent classes yielded a substantial loss in goodness-of-fit, Δ*l*_(*df* = 1)_ = 8.23>*χ^2^_crit_*, *p*<.001, implying that latent classes differed with regard to parameter *s*. Differences between latent classes were observed neither for baseline performance *b* nor for *Goal Ambiguity g*: Levels of baseline performance were comparable across latent classes, *b_SD+_* = .76 and *b_SD−_* = .71 (an equality restriction did not cause a significant loss of fit, Δ*l*_(*df* = 1)_ = 0.2). Both latent classes were affected by higher demands on flexibility and the related breadth of search processes (i.e. an increase in *Goal Ambiguity*) to the same extent, *f_SD+_* = .82 and *f_SD−_* = .78 (again, an equality restriction on the parameters did not cause a significant loss of fit, Δ*l*_(*df* = 1)_ = 0.04).

**Figure 3:**
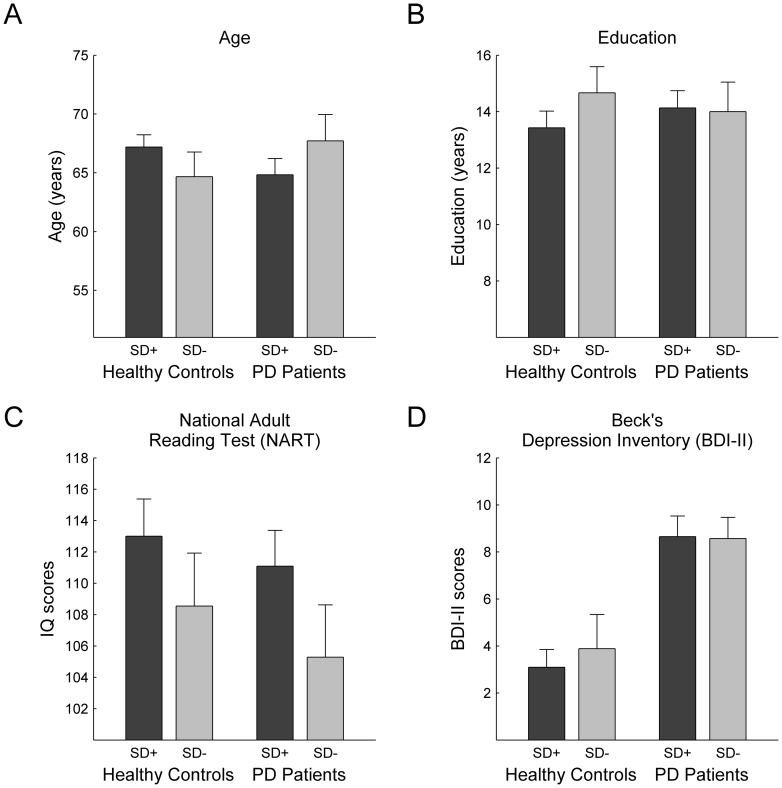
Dissociation of planning disturbances following *PD Diagnosis* and *SD Impairment*. Performance differences between latent classes (panel columns: SD+ vs. SD−) concern the ability to solve problems with high demands on *Search Depth*, whereas PD patients (compared to healthy controls; panel rows) are specifically impaired in problems with a high *Goal Ambiguity*. Note that subjects allocated to the SD− group were not able to solve any problems with a high demand on *Search Depth* at all. Error bars indicate standard error of mean.

For the additional analyses below, individual patients and healthy controls were accordingly assigned to one of the two groups, based on posterior probabilities of latent-class membership. That is, individuals with a higher posterior probability of belonging to the first latent class were grouped together to form the SD+ group; individuals with a higher posterior probability of belonging to the second latent class were grouped together to form the SD− group. In total, 16 subjects were assigned to SD−, roughly corresponding to a quarter of the overall sample. The remaining 44 subjects were assigned to SD+. The grouping factor was henceforth labeled as *SD Impairment* with the two levels SD+ and SD− (referring to the two latent classes as described above). With respect to *PD Diagnosis*, *SD Impairment* was equally distributed in PD patients (7/23 for SD−/SD+) and healthy controls (9/21), a *χ* ^2^ test did not reveal any significant frequency effects between subgroups (*χ* ^2^_(*df* = 1)_ = .341, *p* = .559). In addition, *SD Impairment* groups did not differ with respect to age, years of education, and crystallized intelligence (Table 1; Fig. 3). For PD patients, *SD Impairment* was neither associated with UPDRS scores (*t*_(*df* = 28)_ = .923, *p* = .364) nor with Hoehn and Yahr ratings (*t*_(*df* = 28)_ = .278, *p* = .783) and also neither associated with age at disease onset (*t*_(*df* = 28)_ = .415, *p* = .691) nor with disease duration (*t*_(*df* = 28)_ = .429, *p* = .671). Furthermore, SD− and SD+ classes did not differ in presence/absence of pharmacotherapeutic agents (two-tailed Fisher's exact test for dopamine agonists: *p* = .225, for anticholinergic agents: *p* = .675, for COMT inhibitors *p* = .548, and for MAO-B inhibitors *p* = .153).

**Table 1:** Between-group effects for PD diagnosis and SD impairment.

	Main effects				Interaction	
	PD diagnosis		SD impairment			
Dependent variables	*F*	*p*	*F*	*p*	*F*	*p*
Age	0.04	.844	0.01	.917	2.44	.124
Education	0.01	.994	0.41	.522	0.79	.377
NART	0.69	.410	2.70	.106	0.05	.829
BDI-II	20.91	<.001	0.10	.751	0.15	.698
MMSE	26.25	<.001	5.27	.025	11.78	.001
DRS-2 (AMSS)	6.57	.013	3.93	.052	0.14	.713
CLOX1^a^	4.02	.050	6.27	.015	2.02	.161
CLOX2^a^	0.77	.384	0.09	.760	0.26	.616

*Note.* NART, National Adult Reading Test; BDI-II, Beck Depression Inventory; MMSE, Mini Mental Status Exam; DRS-2 (AMSS), Dementia Rating Scale (age-corrected scores), CLOX1, Executive Clock-Drawing Test (unpromted task); CLOX2, Executive Clock-Drawing Test (copy task).

a N = 59 (data for one PD patient was not available).

### Differential patterns in planning ability

As already inferred from the inspection of parameter estimates (see above), latent classes differed particularly with respect to subjects' average planning performance in problems with high demands on search depth. Subsequent analyses evaluated the effects of latent-class membership on planning performance in relation to the diagnosis of Parkinson's disease. A repeated-measurements ANOVA with between-subject factors *PD Diagnosis* (PD vs. healthy controls) and *SD Impairment* (SD+ vs. SD−), and within-subject factors *Goal Ambiguity* (high vs. low) and *Search Depth* (high vs. low) revealed, as expected, significant main effects for *SD Impairment* (*F*_(1,56)_ = 36.28, *p*<.001), *Goal Ambiguity* (*F*_(1,56)_ = 5.93, *p* = .018), and *Search Depth* (*F*_(1,56)_ = 47.52, *p*<.001), but not for *PD Diagnosis* (*F*_(1,56)_ = 1.01, *p* = .318). These main effects reflect that, apart from PD patients' previously reported problem with ambiguous goal hierarchies, participants also differed in their ability to cope with higher search depths. Interestingly, a highly dissociative pattern was obtained for the two-way interaction effects: *PD Diagnosis* showed a strong trend for an interaction with *Goal Ambiguity* (*F*_(1,56)_ = 3.61, *p* = .063) but not with *Search Depth* (*F*_(1,56)_ = .03, *p* = .868), whereas *SD Impairment* showed a significant interaction with *Search Depth* (*F*_(1,56)_ = 20.74, *p*<.001) but not with *Goal Ambiguity* (*F*_(1,56)_ = .18, *p* = .672). This pattern is illustrated in Figure 3: Firstly, PD patients (bottom panels), but not controls (top panels), were affected by higher in-breadth search demands. Secondly, the SD− subgroups (right panels), but not the SD+ subgroups (left panels), show dramatic effects of an increase in the depth of search demands. None of the remaining two-, three, and four-way interactions reached significance (highest *F* = 1.37, lowest *p* = .247). Thus, the specific planning impairments previously observed in Parkinson's disease and those revealed by the present data-driven classification approach seem to constitute two independent phenomena.

### Additional analyses on clinical tests for dementia and depression

**Figure 4:**
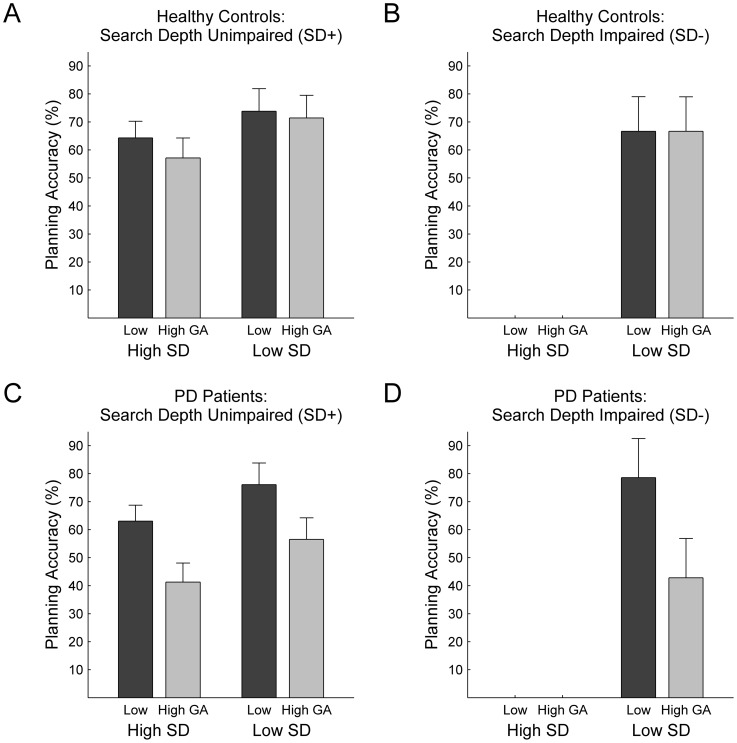
Comparison of latent classes and manifest groups concerning demographic and clinical variables. No differences existed between latent classes with respect to subjects' (A) age, (B) years of education, (C) crystallized intelligence as measured by the NART, and (D) depression ratings as measured by the BDI-II. Panels are partitioned with respect to the two between-subject factors of interest, i.e. *SD Impairment* (SD+ vs. SD−) and *PD Diagnosis* (healthy controls vs. PD patients). Error bars indicate standard error of mean.

**Figure 5:**
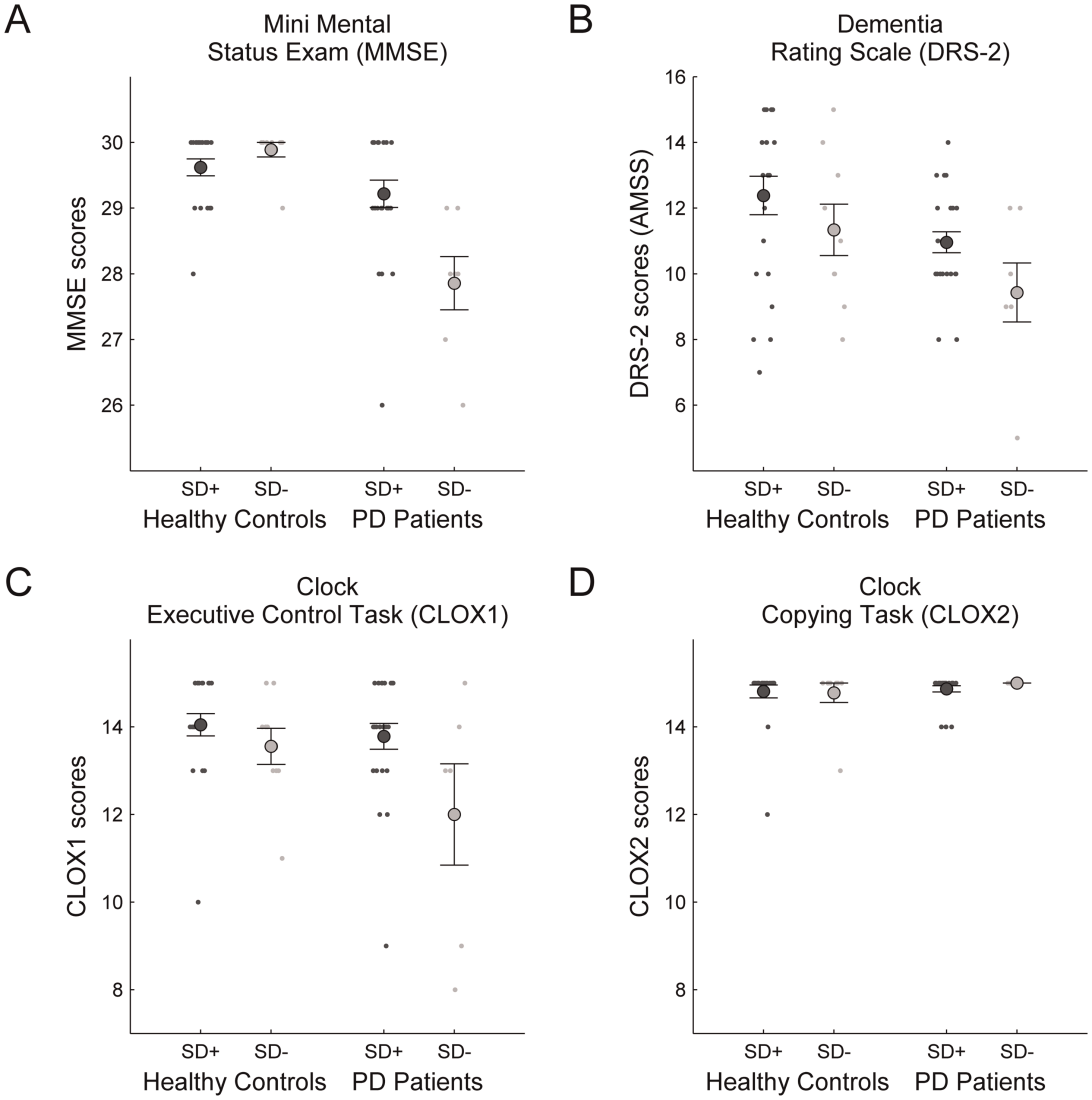
Relationship between latent-class membership and dementia screening ratings. Association of *SD Impairment* with poorer pre-clinical dementia ratings for the (A) MMSE screening test, the (B) more sensitive DRS-2, and the (C) executive control and (D) copying subtests of the CLOX. Panels are partitioned with respect to the two between-subject factors of interest, i.e. *SD Impairment* (SD+ vs. SD−) and *PD Diagnosis* (healthy controls vs. PD patients). Scatterplots illustrate the distribution across individual subjects. Large dots denote group averages; error bars indicate standard error of mean.

Because of the drastic disruptions of planning performance in the SD− group in problems with higher demands on *Search Depth*, further explorative analyses addressed potential clinical markers that might distinguish between the SD+ and SD− groups. As data for clinical assessments of dementia (MMSE, DRS-2, CLOX) and depression (BDI-II) were available, these were entered as dependent variables into separate ANOVAs with between-subject factors *PD Diagnosis* and *SD Impairment*. Results are illustrated in Figures 4 and 5, and inference statistics are provided in Table 1. As reported before, PD patients differed significantly in dementia and depression ratings, albeit deviations were not clinically relevant (cf. [15]). Note, however, that the SD- group also had significantly lower scores on the CLOX1 which measures executive dysfunction and showed a strong trend for reduced DRS-2 scores (see Table 1). Furthermore, an interaction with PD Diagnosis revealed lower MMSE scores for the SD-impaired PD patients. Thus, although on a purely sub-clinical level, *SD Impairment* appeared to be associated with poorer outcomes on screening instruments for dementia.

Recently, cutoff values for DRS-2 scores in PD patients were proposed [32] for a classification into PD-NC (normal cognition), PD-MCI (mild cognitive impairment), and PD-D (demented). According to this recent classification, four controls and two PD patients were classified as MCI (AMSS between 6 and 8), and one PD patient was classified as PD-D (AMSS below 6). One of these control subjects and two of these PD patients belonged to the SD− class. Although DRS-2 classification is purely descriptive and does not represent a clinical diagnosis [32], analyses for the dementia screening measures were repeated only with subjects deemed cognitively normal to preclude the possibility that results of an association with *SD Impairment* were driven by these few cases of borderline cognitive status. The main effect of *SD impairment* on CLOX1 was mildly attenuated (N = 52; *F*_(1,48)_ = 3.60, *p* = .064), but it maintained significance for the MMSE (*F*_(1,49)_ = 10.55, *p* = .002; interaction with PD Diagnosis: *F*_(1,49)_ = 19.98, *p*<.001) and was also significant for the DRS-2 (*F*_(1,49)_ = 5.46, *p* = .025). That is, even when applying more rigorous criteria for normal cognitive status, *SD Impairment* was associated with lower scores on dementia screening tests.

## Discussion

Present re-analyses of the data of McKinlay et al. [15] aimed at testing for possible sample-immanent heterogeneity of responding to increased demands on the breadth versus depth of searching ahead during problem-solving on the Tower of London (ToL) task. By applying a latent-class model-based approach [19], [20], further within-group heterogeneity in planning performance of Parkinson's disease (PD) patients and healthy controls could indeed be identified. Performance of the overall sample could not be accommodated by a single class. Instead, it was best described by a set of two latent classes differing with respect to increased demands on the depth of anticipatory look-ahead processes as imposed by higher levels of search depth. While the SD+ class was characterized by a moderate drop in performance on problems with high search depth (approximately 80% of baseline accuracy), the SD− class demonstrated a dramatic decrement in planning accuracy with performance dropping to floor levels.

Given the well-known cognitive impairment in patients with PD [1], [2], one might expect latent classes to largely mirror the manifest groups of PD patients versus healthy controls. However, there were no frequency differences between PD patients and healthy controls concerning membership in SD+ and SD− groups, respectively. That is, the differential impairment of performance on problems with high search depths was independent of PD diagnosis. Furthermore, experimental manipulations of the breadth versus depth of planning processes interacted differentially with manifest groups and latent classes in affecting subjects' planning performance. Higher goal ambiguity impaired performance in PD patients compared to healthy controls, but did not show any association with latent-class membership. Conversely, variations in search depths of problems did not interact with manifest group assignment but with membership in latent classes, revealing that subjects of the SD− group were selectively impaired in problems posing high demands on in-depth search processes. Thus, present re-analyses did not only identify sample-immanent heterogeneity in planning performance hitherto undetected by conventional group-based approaches [15]. They also indicate that deficits in dealing with high demands on the depth of look-ahead search processes can be clearly dissociated from deficits in the breadth of searching ahead even within a single task, each giving rise to specific planning impairments.

As expected, PD pathology was found to be associated with a selective deficit in problems requiring the flexible organization of mental representations in identifying the optimal sequence of final moves, hence reflecting results of the original analysis [15] and further corroborating the notion of compromised cognitive flexibility in PD patients [1]. In light of previous inconsistency regarding PD-related impairments on the ToL [3]–[8], carefully manipulating the demand for cognitive flexibility in future studies might therefore help to shed light on the exact nature of planning deficits in PD and on their relation to different stages of disease progression.

Most intriguingly, disproportionate disruption of planning performance by higher search depths of problems was associated with poorer ratings on clinical screening instruments for dementia. In other words, failure to cope with increased depth of anticipatory steps along the solution path during planning was related to signs of beginning cognitive decline in both PD patients and healthy controls. It has been often reported that MCI is a prevalent concomitant syndrome in PD [33], [34] with high conversion rates to dementia [35], so that PD is accompanied by full-blown dementia in as much as 25% to 40% of patients [36],[37]. In line with this, patients' MMSE and DRS-2 scores were significantly lower compared to controls and CLOX1 scores showed a strong trend for a similar difference. Importantly, however, despite these overall signs of cognitive deterioration in PD patients, different latent-class membership within the PD group was associated with significant differences in dementia ratings. This association also held up for more rigorous assessments of normal cognitive status according to DRS-2 cutoff values (cf. [32]). That is, beyond deficits in cognitive flexibility and signs of increased cognitive decline related to PD pathology, search depth-related impairments independently accounted for further systematic variance. It has been previously found that beyond a general deficit of PD patients in ToL performance, demented patients, indicated by MMSE scores below the normative cutoff value, performed even worse than non-demented PD patients [3]. The general difference in performance between PD patients and healthy controls still held up after exclusion of demented patients [3], which suggests that executive deficits associated with cognitive decline are independent determinants of planning performance in PD patients. Current results extend this finding in demonstrating that, first, signs of cognitive decline are associated with a specific planning impairment rather than overall performance decrements and, second, that this association is not only valid for PD patients but also for healthy controls (control subjects were not screened for dementia by Culbertson et al. [3]). Therefore, the detrimental impact of increased demands on the depth of searching ahead is informative of cognitive impairment related to dementia in patient as well as normal populations, possibly indicating a pre-clinical stage of the disease process. This is in line with the fact that, besides profound memory impairments, Alzheimer's disease (AD) and related forms of dementia are also known to affect executive functioning and, specifically, planning abilities, even in early stages of the disease (for a review, see [36]). Accordingly, the ToL has been proven to be a sensitive instrument for detecting executive deficits present in AD [38]–[40] as well as in frontal lobe dementia [41]. Furthermore, the ToL has been successfully employed to identify demented patients, with overall accuracy yielding a sensitivity and specificity of more than 75% in distinguishing patients with moderately progressed dementia from controls [39], and has been shown to differentiate between patients with AD and fronto-temporal dementia with an accuracy of nearly 80% using artificial neural network modeling [42]. While patients in clinical stages of dementia are especially prone to decreased overall accuracy and excessive rule-breaking [40] ( see also [41]), here it was revealed that subjects who have not yet developed clinical signs of dementia do not solve fewer problems overall but selectively fail when problems demand an increased depth of anticipatory look-ahead processes for identification of move interdependencies and resultant sequences. Caution is however warranted in interpreting this association of ToL performance with sub-clinical signs of dementia as it is based on post-hoc analyses rather than on a prospective study design. It cannot be ruled out that differences in dementia ratings reflect inter-individual differences in general mental capacity rather than the onset of the dementia process, given that participants were not followed up longitudinally. This restriction notwithstanding, present explorative analyses might provide a first insight into the origins of different planning impairments associated with executive dysfunctioning in dementia and Parkinson's disease and might be the first indication of a putative underlying double dissociation of deficits regarding the breadth versus depth of planning processes.

The dissociation between planning deficits in the depth versus breadth of searching ahead found here, putatively related to reduced cognitive stability and flexibility, accords well with the concept of the latter's neurochemical correlates as two independent, but functionally reciprocal mechanisms [1], [9] (see also [11]). No inferences can however be drawn from the present results about the neural locus of these planning deficits. Recently, we found that variations in goal hierarchy of three-move ToL problems were associated with increased activity of the left dorsolateral prefrontal cortex [43] (see also [44]), which seems to argue against a striatal correlate of cognitive flexibility as proposed by Cools [1], [9]. However, higher goal ambiguity in three-move problems does not entail the same cognitive processes as in more complex five-move problems applied here (see [45]), so that a direct comparison of the neural underpinnings of cognitive processes related to simpler versus more complex ToL problems is unwarranted. Hence, the neural locus of deficits in cognitive flexibility and stability within the fronto-striatal dopaminergic system and their relation to PD pathology have yet to be identified.

In conclusion, present re-analyses using a latent-class model-based approach provided a valuable extension to the original study [15] in identifying further systematic variation in planning performance on the ToL. Overall performance is thus often less informative than what can be revealed by considering important task parameters and by using model-based analyses to identify systematic heterogeneity that would otherwise remain undetected by group-based approaches (cf. [19], [20]). Here, it was possible to delineate dissociable impairments in the breadth and depth of search processes during planning within a single task of visuospatial problem solving. These specific planning impairments seem to differentiate between distinct clinical phenotypes. Whereas Parkinson's disease pathology is associated with impaired in-breadth search processes, possibly indicative of reduced cognitive flexibility, sub-clinical signs of dementia may be related to deteriorated depth of searching ahead during planning. Further research employing prospective study designs is needed to investigate this putative pattern of differential planning impairments and could promote an enhanced understanding of the specific cognitive deficits associated with prevalent neurological disorders such as Parkinson's disease and dementia.
